# Anomaly Detection for the Centralised Elasticsearch Service at CERN

**DOI:** 10.3389/fdata.2021.718879

**Published:** 2021-11-16

**Authors:** Jennifer R. Andersson, Jose Alonso Moya, Ulrich Schwickerath

**Affiliations:** ^1^ Department of Information Technology, Uppsala University, Uppsala, Sweden; ^2^ Department of Computer Science and Engineering, Universidad Carlos III de Madrid, Leganes, Spain; ^3^ IT Department, CERN, Geneva, Switzerland

**Keywords:** anomaly detection, elasticsearch, LSTM, DNN, machine learning

## Abstract

For several years CERN has been offering a centralised service for Elasticsearch, a popular distributed system for search and analytics of user provided data. The service offered by CERN IT is better described as a service of services, delivering centrally managed and maintained Elasticsearch instances to CERN users who have a justified need for it. This dynamic infrastructure currently consists of about 30 distinct and independent Elasticsearch installations, in the following referred to as Elasticsearch clusters, some of which are shared between different user communities. The service is used by several hundred users mainly for logs and service analytics. Due to its size and complexity, the installation produces a huge amount of internal monitoring data which can be difficult to process in real time with limited available person power. Early on, an idea was therefore born to process this data automatically, aiming to extract anomalies and possible issues building up in real time, allowing the experts to address them before they start to cause an issue for the users of the service. Both deep learning and traditional methods have been applied to analyse the data in order to achieve this goal. This resulted in the current deployment of an anomaly detection system based on a one layer multi dimensional LSTM neural network, coupled with applying a simple moving average to the data to validate the results. This paper will describe which methods were investigated and give an overview of the current system, including data retrieval, data pre-processing and analysis. In addition, reports on experiences gained when applying the system to actual data will be provided. Finally, weaknesses of the current system will be briefly discussed, and ideas for future system improvements will be sketched out.

## 1 Introduction

Due to increased interest from its user community, CERN-IT decided to offer a new, managed and centralised service for Elasticsearch^1^ ^,^ ^2^. As part of the initial service deployment, a detailed internal monitoring scheme was implemented, allowing service managers to detect and debug issues early on. Since one of the main customers from the start was the central monitoring infrastructure itself, a dedicated Elasticsearch cluster was created for the monitoring of the service to avoid circular dependencies. Over time, the internal monitoring scheme was enriched by detailed dashboards and visualisations for a variety of relevant metrics. This allows the service managers to debug any issues with the service. The historical record of the monitoring data can also be directly used to train an anomaly detection system to detect issues in an automated way.

An initial attempt to extrapolate the future state of the clusters based on their history was made back in 2016 and completed in 2017 (Moya, 2018). Based on the experiences gained during this exercise, as well as profiting of the increased monitoring data accumulated over the years, the approach was reviewed in early 2019, and further developed during that year, resulting in the current anomaly detection system used in production. In the following sections, a brief description of the service and previous attempts to implement an anomaly detection system to detect issues in the Elasticsearch Service will be given. Data retrieval, flow and pre-processing will be covered, along with an overview of the neural network training process and an analysis of the final performance. Finally, examples of performance on real monitoring data are given. The paper will conclude with the mention of known limitations and future work. It is important to mention that the focus of this project has been less on the optimisation of the analytics part but on the usability in practice.

## 2 CERN Centralised Elasticsearch Service

The Centralised Elasticsearch service provides a set of independent Elasticsearch installations, also called clusters. The details about the service can be found in (Saiz and Schwickerath, 2020). A sophisticated access control system allows for sharing individual clusters between different user communities, while ensuring that they remain hidden from each other, see (Schwickerath et al., 2019) for more details. In this way, each cluster has one or more entry points that can be used by entirely different user communities, thereby allowing for efficient sharing of the available resources. All the client traffic goes through a web proxy which gives additional information about access patterns, timings and return codes.

## 3 Anomaly Detection Development

### 3.1 Auto-Encoder and Classifier

A first attempt to use machine learning methods to predict issues in the Elasticsearch service was made in 2016 by a technical student (Moya, 2018). This was based on a two step approach aiming to extrapolate cluster states into the future, consisting of an unsupervised deep auto-encoder (Said Elsayed et al., 2020) architecture followed by a supervised classifier predicting the future state per cluster in the next 5–10 min. The classification step was used on events pre-selected by the auto-encoder, and tried to predict the future cluster health from the set of metrics in use. It was trained using the Elasticsearch cluster health state (“operational,” “affected” and “down”), as this step requires annotated data. Due to the different use patterns of the different clusters, the system was applied to each cluster independently.

The approach suffered from a number of issues:

• Lack of statistics for the classifier: while there was a lot of available cluster data in fully healthy states, the number of “affected” and “down” states of interest for service managers, as reported by Elasticsearch, became rare while the service maturity was improving, making it impossible to re-train that part of the system due to lack of statistics.
• With increased maturity of the service, users reported more and more on issues which did not affect the cluster health at all, making the classifier effectively irrelevant as these do not affect shard allocations.
• The auto-encoder suffered from frequent convergence problems, specifically for more complex clusters.
• It was not possible to apply the system to new clusters as this required re-training of the particular cluster with enough statistics.
• Cluster usage patterns changed over time due to changing user activity, making frequent re-training necessary.
• Unpredictable user activity resulted in many false positive alarms after the auto-encoder step.

Due to these issues, the classifier was dropped early on, leaving only the data retrieval and the auto-encoder. The remaining system was still useful to detect issues by directly checking the anomaly scores defined by the auto-encoder. While moving to newer versions of Elasticsearch, there was a need to retrain the auto-encoder for new clusters, which turned out to be very time-consuming and very unreliable due to the convergence issues.

### 3.2 Re-implementation Based on Long Short Term Memory

In 2019 the whole system was reviewed, including the goal of the anomaly detection system as such. Rather than trying to predict the future cluster state, the focus turned to guiding service managers on where to pay attention first, with the ultimate goal of providing a single dashboard to look at several times per day to spot issues as they appear, before users do. Rather than using a deep feed-forward neural network architecture, it was decided to go for an unsupervised model based on Long Short Term Memory (LSTM) networks (Hochreiter and Schmidhuber, 1997) to address the convergence issues. LSTMs are powerful recurrent neural network architectures capable of capturing long-term time dependencies, and have been used successfully for time series anomaly detection in the past, see for example (Said Elsayed et al., 2020). This could increase the possibility of capturing service issues that build up over time. An overview of the current status of this approach was written in mid-2019 (Andersson, 2019). In addition, a simple moving average approach was introduced as a traditional method which does not require any training and which has no free parameters (see Section 5.1). This was done with the objective of having a simple prediction method to which the LSTM results could be compared.

### 3.3 Generalised Model

Finally, the model was extended to all clusters, allowing for less time to accumulate training data, and opening up the possibility of immediately applying the system to newly created clusters. This was possible by a thorough review of all used metrics, replacing those which were cluster-dependent by similar metrics with the same information content, but with the same range for all clusters. As an example, the number of data nodes in a cluster was replaced by the relative node count with respect to the previous sample, and the free disk space was replaced by the percentage of free available space.

## 4 Description of the Anomaly Detection System

The basic idea is the following. Every Δ*t* = 5 min, each cluster reports its state as a vector *y* (*t*_0_) of dimension *n*, where *n* is the number of metrics used to detect anomalies. If *m* is the number of time steps in the past to be used, then the samples *y* (*t*_−*m*_) to *y* (*t*_−1_) are used to make a prediction *Y* (*t*_0_) for the current time *t*_0_.

The final anomaly score *S* (*t*_0_) is then defined as the mean squared error of the prediction, *Y* (*t*_0_), and the ground truth provided by the measurements, *y* (*t*_0_):

$$S\left(t_{0}\right)=\frac{1}{n}\overset{n}{\underset{i=1}{\sum }}{\left(y_{n}\left(t_{0}\right)-Y_{n}\left(t_{0}\right)\right)}^{2}$$ (1)

where n is the number of metrics. Note that the normalisation factor $\frac{1}{n}$ is actually of no importance for the anomaly detection itself. In addition, in case this value is larger than expected, the differences *y*_*i*_ (*t*_0_) − *Y*_*i*_(*t*_0_) contain valuable information about the potential origin of the issue.

**FIGURE 1:**
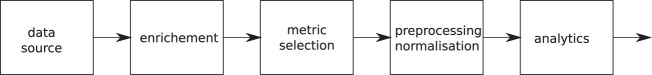
Processing pipeline: independently of the source, the data is processed by the same pipeline to ensure consistency.

The system will be described in the following section, starting with an overview of the information flow, and then going into more details. Figure 1 shows the processing pipeline. The data source can be historical data or fresh data flowing in from the system. The processing is ensured to be identical during training and evaluation of fresh data. All data is first enriched in the sense that other, derived values are added to the set, like derivatives of input variables. After that, a subset of metrics from Elasticsearch internal state data, log-files and machine monitoring, as indicated in Table 1, are selected and preprocessed if needed. Before entering the analytics part, it has to be normalised in a well-defined way.

**TABLE 1:** Sources and classes of input metrics. During the enrichment steps, averages, maximum and minimum values as well as changes with respect to the previous time step are calculated, and a subset of these are used in the final model.

Information source	Metric groups
host monitoring metrics	• CPU usage
	• memory usage
	• disk usage
	• swap usage
Elasticsearch internal metrics	• breakers related metrics
	• thread pool related metrics, namely for search and write requests
	• java virtual machine metrics
	• garbage collectors
	• index, shards and document counts
	• index and shards field, replication, storage size and segment information
	• query cache usage
	• node counts
web server log files	• bytes transferred
	• response time
	• exit codes, aggregated over ranges, e.g. number of 4xx codes
Elasticsearch log files	• connection exceptions
	• deprecated requests
	• water mark warnings from Elasticsearch
	• java errors
	• node number change events (left, joined)
	• warnings, e.g. for too large requests

### 4.1 Implementation of the Processing Pipelines

**FIGURE 2:**
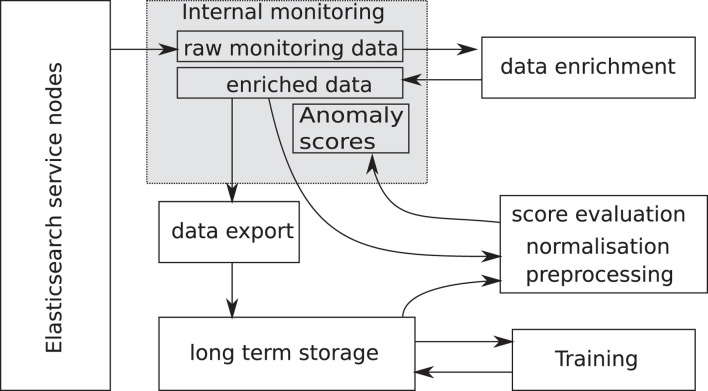
Information flow for the anomaly detection system.

The actual implementation of the processing pipeline is shown in Figure 2. The internal monitoring system serves as data source, where all metrics are directly accessible from the monitoring dashboards. A subset of metrics is periodically extracted, joined and enriched according to the previous description, and written back to the monitoring system. The enriched data is dumped once per day on a mass storage system, see for example (Bitzes et al., 2020), for later use during the training. In this way, the training can be done entirely externally to the service itself. The training procedure uses this historical daily enriched data and produces both the model parameters and normalisation information, which is needed to pre-process fresh data before feeding it into the model for evaluation. It is possible to run several models (e.g. one for production and another one for quality assurance) in parallel. Evaluation of the model itself happens every 5 min on fresh data from the monitoring system. Scores, as well as the contributions of each input, are written back into the monitoring system and visualised on a dedicated dashboard.

### 4.2 Data Processing

The data retrieval is performed every Δ*t* = 5 min. At this step the goal is to retrieve as much information from the system as possible and to keep it as generic as possible. The selection of metrics is done in a later step in the pipeline, which is model specific. The idea is to keep the system as flexible and open as possible to the application of different analytics steps later on. Since monitoring data is stored on Elasticsearch, this step implies aggregation of data in the given time interval Δ*t*, including the calculation of sums, averages, minimum and maximum values or counts. Input data comes from different sources. A rough classification of metrics and their sources can be found in Table 1.

### 4.2.1 Logging Information

Log files typically contain human readable text with very valuable information about the state of system, including warnings and errors. While convenient for humans, the analytics part expects metrics which are numbers. Therefore, log files require a special treatment. Each log entry is parsed with regular expressions and categorised. The number of entries in each category per time interval is then used as a metric, each category corresponding to a different metric. This very basic approach is good enough to catch the most frequent failure modes. More sophisticated methods could help, in particular for cases where log file entries change between different versions of the software being monitored, or to catch new entries. This, however, is not part of this paper, and is on the road map for possible future improvements.

### 4.2.2 Data Enrichment

Data enrichment adds new metrics to the set of metrics by deriving them from the input data. This implies:

• Calculation of derivatives, approximated by the difference between the current and the previous sample. As an example, the absolute number of accesses to a system on its own can vary a lot for different use cases of a cluster, and may vary as well over the lifetime of the cluster due to changed user behaviour. Short-term changes of this number though are less sensitive to the use case, cluster and slow changes in time.
• Conversion of absolute numbers into relative numbers. As an example, the raw data contains the total and the used disk space. Since different clusters have different size, they are not comparable, while the relative usage of the disk in percent is a metric which is comparable across all clusters.

### 4.2.3 Metric Selection

After enrichment, the data set contains about 400 metrics. Out of the enriched data set, only a subset is actually useful. For example, absolute values which are cluster specific are not useful for further analysis and should be dropped from the pipeline. This also reduces the dimensionality of the problem to be solved in the analytics part later on. Spearman’s rank correlation coefficients were calculated on the set of input metrics of the original model to reduce dimensionality. The current model contains 224 input metrics. The identification and elimination of cluster dependent input metrics is the key to obtaining a generalised model that works for all clusters, including new clusters previously unseen by the system.

#### 4.2.4 Chunking of Input Data

Chunking up of data is only required to prepare the historical data for training. It takes place after the metric selection and before the data pre-processing in Figure 1 when the training and validation sets are defined. In this step, the whole training data set is chunked up into smaller time sequences. The reason for chunking up the data in time is that the behavior of the clusters evolve over some time. After users have asked for a new cluster, it will initially be empty. Then users will start to experiment with it, more users may join in over time, and the load increases. Therefore, the samples for each cluster become a function of time. By randomly picking different periods of time for both training and validation, it is possible to at least partly compensate for this effect. Samples in the validation set are neither used for the determination of meta parameters for normalisation nor for the training itself, but only for validation.

#### 4.2.5 Pre-Processing and Normalisation

The next step is pre-processing and normalisation of the incoming data. Pre-processing involves detection and repair (if possible) of bad or missing data samples, identification of clear outliers, determination of the skew of the inputs, and taking the log of very much skewed input metrics. In this step a Principal Component Analysis (PCA) has been considered to speed up training and reduce the problem dimensionality, which is relatively large. This step is ultimately discarded in the pre-processing pipeline due to the risk of information loss increasing the rate of false negatives. Most input metrics are sensitive to rare failure modes only, and hence, the related metrics will usually be constant. If these metrics change, however, this is something the service managers will need to know about, and therefore these metrics must not be removed from the inputs. The training speed is less of an issue in this specific use case, since retraining of the network is only required if the set of inputs has changed. This typically correlates with significant changes in the monitored service which occur rarely, at a rate of at most two to three times per year.

Next, the data is further processed and normalised. Centralisation using the absolute maximum of each metric, as estimated from the training data set, was found to be too sensitive to outliers which had to be removed before proceeding. Standardisation generally provided better results in this respect and avoided having to remove outliers from the data set prior the training. The required mean values and standard deviations are again retrieved from the training data set only.

The last step in the pre-processing chain is normalisation of the input data. Standardisation (Géron, 2017) is used to normalise the data such that it has a mean value of zero and unit variance. The evaluation of the scores following Eq. 1 requires the model output, *Y*, to be on the same range as the input data, *y*. In many deep learning models, the output layer of the network uses a sigmoid function restricting the outputs to take on values between 0 and 1. Therefore, the values from the system state must be normalised to match that range, or the predicted values cannot be compared to them.

**FIGURE 3:**
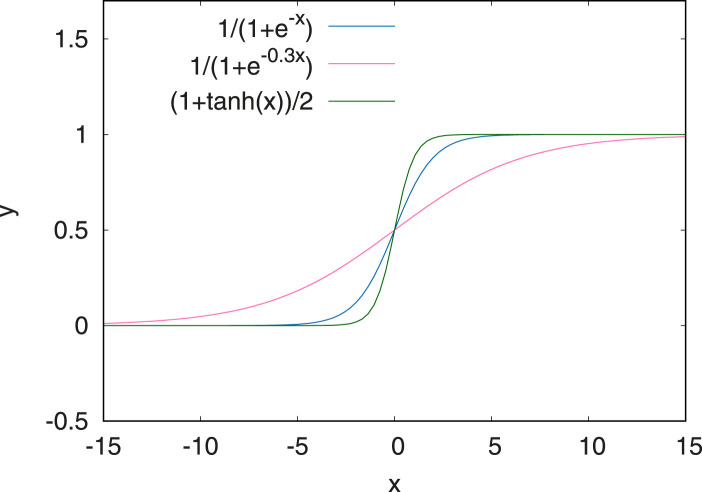
Normalisation functions tested for the anomaly detection. The first one in blue corresponds to a Sigmoid function. The modified sigmoid function (in red) has the advantage of being more linear and flat around zero.

To achieve this, three different options using a non-linear normalisation approach have been explored. These are shown in Figure 3. Both the standard Sigmoid function and the tanh are fairly steep around zero while the modified Sigmoid function $y=\frac{1}{1+e^{-0.3x}}$ only maps extreme outliers close together to zero or one. Using the modified Sigmoid function showed the best performance in a parameter scan and was thus selected. The additional factor of 0.3 was chosen based on a visual inspection of the input data. It is a free parameter which can be further tuned through an extended parameter scan in a future study.

#### 4.2.6 Training Set Preparation

Training uses data across all clusters except those which underwent major changes or which had known larger anomalies during the phase used for training. For the remaining clusters, already identified clear outliers are removed to ensure that these are not considered as normal operation for the neural network.

For the model presented in this paper, 140 days of recorded data were used, sampled every 5 min from 29 selected clusters, corresponding to a total of about 1.2 million data samples. Half of these were used for training, the other half for validation.

## 5 Models

The analytical models which have been tested so far will be described in the following sections.

### 5.1 Moving Average

A simple method which does not require any retraining and adapts itself to the data is to calculate a moving average. The pre-processing pipeline is identical to the one for the LSTM network. The prediction for each cluster is then defined as the average

$$Y\left(t_{0}\right)=\frac{1}{m}\overset{-m}{\underset{i=-1}{\sum }}y\left(t_{i}\right)$$ (2)

The anomaly score is then calculated as in Eq. 1. This very simple approach already catches many of the anomalies in the system. Since it comes with zero free parameters after fixing the time window length to 1 day, it can be applied to each cluster separately, and directly to the incoming data. A caveat of this approach is that it automatically adapts to the current situation. So, if a problem builds up and stays for some hours, it will soon no longer be reported on. Moreover, when the issue goes away, the method will complain again.

The moving average is not capable of catching any correlations between the input variables and thus is less performant for clusters with more complex usage patterns.

### 5.2 LSTM Networks

As mentioned in chapter 3.1, the auto-encoder network suffered from convergence issues and was difficult to train. RNN networks are better suited for time series, and specifically LSTM (Hochreiter and Schmidhuber, 1997) networks address convergence issues. Moreover, LSTMs are designed to capture long-term dependencies, and are therefore well-suited for time series prediction, which matches our use case. This is why this architecture has been chosen.

For the latest model currently in production, about 5 months of historical data has been used. A set of 224 metrics has been selected from the raw data set. The training sample contains data from all clusters, ensuring that data in the time sequences is not mixed up between the different clusters.

The full historical data was chunked up into a total of 20 subsets, and randomly assigned to equally sized training and validation data sets. In previous studies multiple possible network layouts have been evaluated. Using several layers did not improve the performance significantly, but came at the cost of significantly increasing the number of free parameters. Other network architectures investigated included a feed-forward layer as input or output layer, or both. While increasing the number of free parameters, the gain in performance on the validation set was not significant. Using other RNN variants like Gated Recurrent Units (GRU) (Chung et al., 2014) was outside the scope of the current study but is on the roadmap for possible further improvements.

In the final model, a single LSTM layer with 288 time steps is used, corresponding to a full day of historical data. There are roughly 400,000 trainable parameters in the model.

The Keras/Tensorflow^3^ implementation of LSTMs is used. In order to speed up the training process and make use of GPUs, the CuDNNLSTM implementation is used, with Adam (Kingma et al., 2015) as optimizer with a learning rate of 0.001 and a decay rate of zero. The mean squared error is used as loss function.

After the model has been learned, it is converted back into a regular Tensorflow LSTM such that the evaluation can be done on any CPU only based node.

**FIGURE 4:**
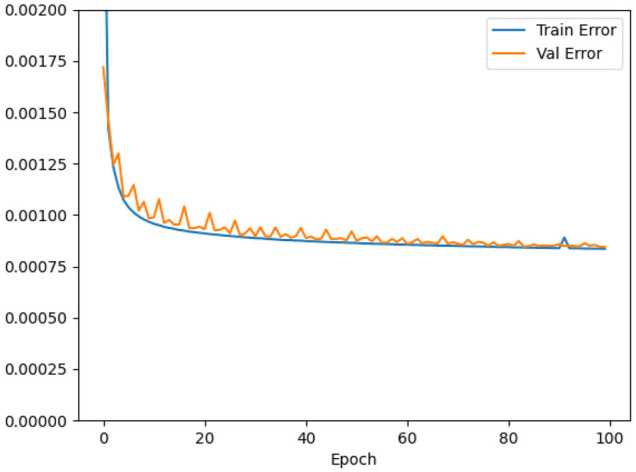
Loss as a function of the number of epochs.

Figure 4 shows the loss during the training as a function of the epoch. The validation loss follows the training loss well. The validation error drops rapidly already after a few epochs and then continues to slowly decrease. The training was interrupted statically after 100 epochs. It should be noted that the error converges to a value above zero. This behaviour is not unexpected since the training sample still contains unpredictable data caused by random user activity.

Training time using a NVIDIA GeForce RTX 2080 with 12 GB RAM takes about 19 h. This could possibly be reduced by tuning the model parameters. This is, however, not considered worth the effort, as re-training is usually only required if the input metrics have changed which recently only happened every few months. Apart from Adam, other optimizers, namely SGD (Murphy, 2022) and RMSprop,^4^ have been investigated. Using Adam with the chosen learning rate showed the best convergence behaviour for this specific use case.

## 6 Results

The LSTM based model solves the convergence issues observed in Section 3.1. Convergence and generalisation is generally good to excellent. The fact that the training error stays above zero is expected due to the partially unpredictable data.

In practice, the single generalised model is applied to each cluster individually every 5 min, and the scores are written back to the monitoring system for use by the service managers.

**FIGURE 5:**
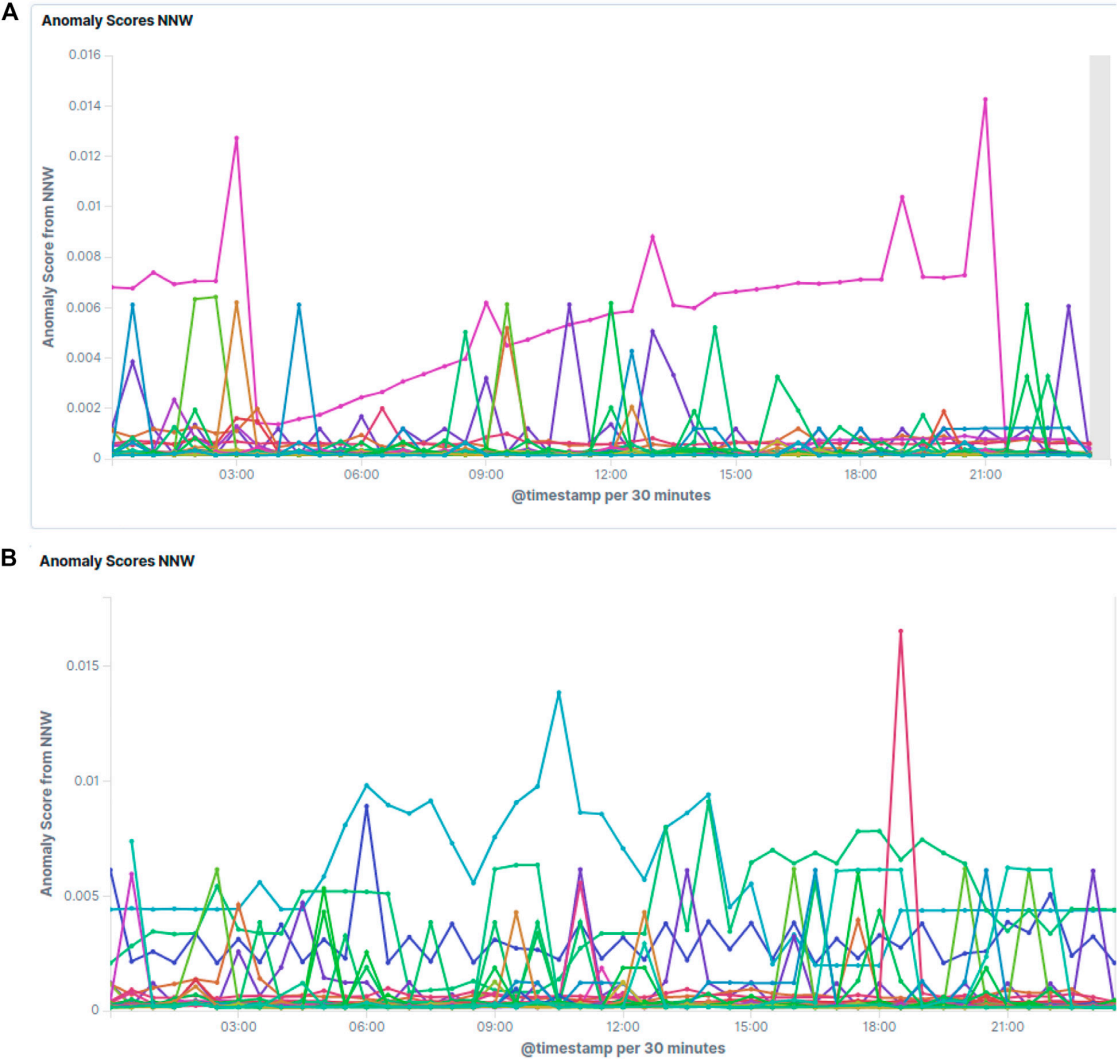
Screenshots of the raw anomaly scores as estimated by the LSTM network. Each line corresponds to a different Elasticsearch cluster. Lower values are better, meaning that the prediction matches the actual value better. Clusters with scores sticking out above the others over longer times of periods or with increasing scores over time need attention. Figure 5A normal conditions with some noise and one cluster with an issue building up, and Figure 5B a typical full day showing several issues to be checked.

Figure 5 shows some examples of the resulting anomaly scores on real data. Random user activity as in 1) turns up as sharp, usually small spikes, which occur more often during working hours and recover immediately. Increased activity during the night can be explained by the creation of new indices or analysis of the data collected during the day by the users. Unless these spikes become very frequent and high, no special attention is required. In some cases users have to be contacted to optimize their usage of the system though. If the score for a specific cluster builds up slowly over time as can be seen in Figure 5A, or if it jumps up and stays at a high level, the cluster in question deserves expert attention. Figure 5B shows the anomaly score evolution during the course of a full day for all clusters, indicating several issues requiring expert attention.

While looking at the raw anomaly scores, which are very fine-grained with samples every 5 min, it is sometimes better to aggregate them over a longer period of time. Doing that has the advantage of smoothing out peaks caused by random and unpredictable cluster activity, instead focusing attention on longer ongoing issues which need expert attention.

**FIGURE 6:**
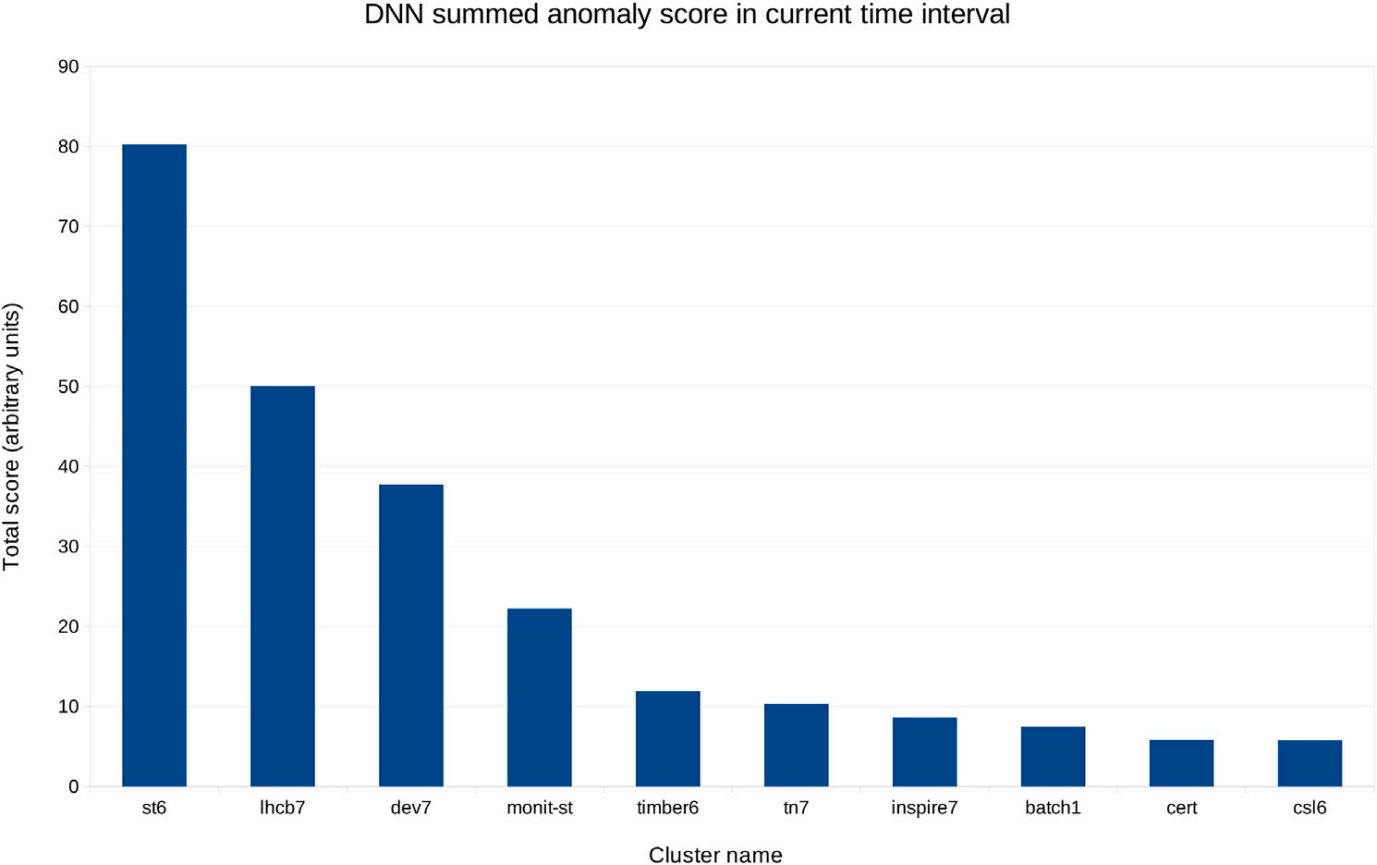
Aggregated score for the same day as in Figure 5, from 8am to 12pm, in decreasing order.

Figure 6 shows the aggregated scores per cluster in a 4 h interval, in decreasing order by cluster. This view is updated every 5 min, showing the status for the last 4 h at any given time, and can thus guide experts in deciding where to focus their attention.

**FIGURE 7:**
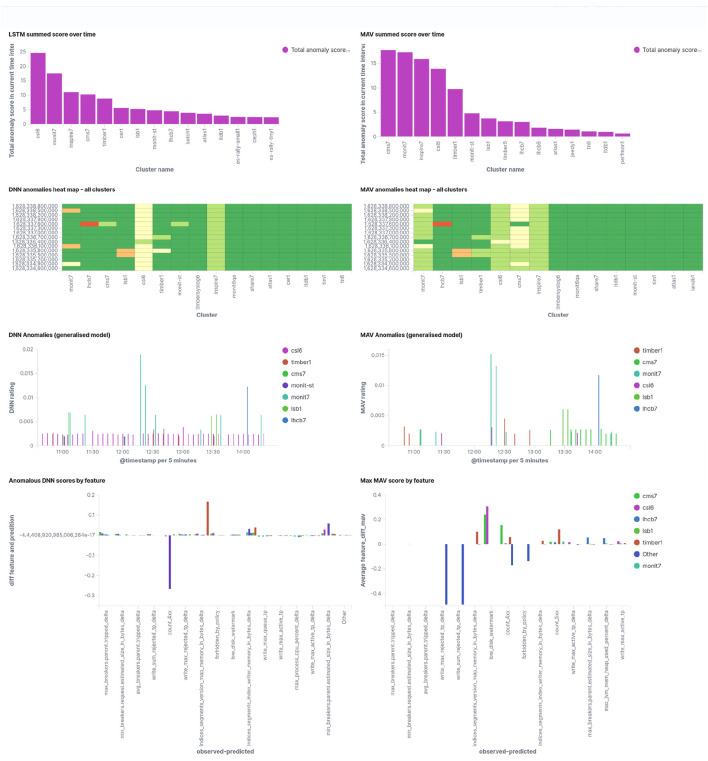
Screenshot of the anomalies overview page for service managers, showing the LSTM **(left)** and the moving average **(right)** results for the last 4 hours of operations. The upper row shows the accumulated anomaly score, giving an indication of what to look first. The second row shows the last scores in 5 min steps, color encoded. Non-green bands indicate ongoing issues. The third row shows all anomaly scores by time which exceed a specific threshold, and the fourth row shows input metrics with the largest deviation from the predicted value, thus giving an indication of what may be wrong.

Finally, Figure 7 shows a screenshot of how the service managers will see this. Note that the screen shot has been taken at a different time than the data represented in Figure 6. Scores from both the LSTM and the moving average are given, as they are complementary since the LSTM predictions are based on reference period (used for training) for all clusters, while the moving average is cluster specific, and adapts rapidly to ongoing issues such that they will completely vanish from the picture if they have been going on for 24 h or more already.

Comparing LSTM results and moving average predictions it can be noted that in particular for simple and frequent cases the moving average does fairly well. Not surprisingly, anomaly scores for different clusters differ between the methods. Long standing issues (for example, during weekends) are no longer detected by the moving average after 24 h or more, and turn back into false positive anomalies when the actual issue has been fixed. Nevertheless, the moving average results are kept on the service managers dashboards, as they are complementary to the LSTM estimations.

## 7 Experiences in Daily Operations

The described system has been in production for about a year and a half. Since January 2020, the system has been retrained seven times, due to model improvements and to address false negatives which turned up during daily operations and which required additional metrics to be added to the model.

In daily operations, the anomaly detection system is a useful extension of the already existing and maintained detailed service monitoring. Its strength is that it only takes a few seconds between flagging an anomaly for a particular cluster and identifying possible reasons for the anomaly. A follow-up is then needed by visiting related detailed dashboards in the standard monitoring system to get to the bottom of the issue, while filtering for the specific cluster for which the anomaly has been seen. Quantifying the amount of time this spares service managers in their daily work is difficult but the current system significantly reduces the workload and streamlines the working process.

The bulk of the detected cases, in the order of 80%, are related to user errors, for example wrong credentials, bad clients, or simply users filling up the available storage space. Even if these do not cause a problem for the service itself, they are easy to overlook in the standard monitoring and have frequently been missed out in the past. They can cause data loss for the users though, and users appreciate being notified of these cases.

Feedback on the anomaly detection system from users and other service managers who make use of it is very positive in the sense that it makes their daily operations easier.

## 8 Limitations and Future Work

The optimisation of the current system has been done mainly by hands-on experience while using it to detect service issues. A currently still missing feature is the possibility to annotate detected anomalies, which would allow for quantitative estimation of the performance of the system by service experts confirming or disproving detected anomalies. Additional information about the anomaly type would be beneficial in that step, as this would allow for grouping the detected anomalies into larger categories, enabling further prioritisation of actions to be taken by the service managers. Examples of such categories would be: user errors, disk space issues, system I/O issues, etc. Note that his is different from the original approach which used a classification network based on the Elasticsearch internal cluster status. Depending on the anomaly type, it may also be possible to trigger automatic corrective actions where possible.

An ever ongoing iterative process is the selection of the input variables. While using the system in production, a few false-negative events have been identified, for example by users complaining about performance issues which were not seen by the anomaly detection system. These cases could be traced down to missing sensitivity in the input data, and they have been resolved by adding metrics with sensitivity to those events and retraining afterward. Similarly, the number of false positives has recently been significantly reduced by removing three noisy input variables from the model, among them the current cluster state reported by Elasticsearch. The latter can change quickly if there is heavy indexing activity, as it indicates if all data has been written to disk or not.

On the other hand, the dimensionality of the input space, O (200), is still very high, and it is likely that the performance of the system can be further improved by reviewing the feature extraction process and eliminating input features that have not been observed to contribute to any anomalous events in the past, accepting the increased risk for false negatives on rare anomalous events sensitive to those particular input features.

## 9 Conclusion

Using both traditional methods and machine learning techniques, a robust and fully self-supervised anomaly detection system has been implemented for the Centralised Elasticsearch service at CERN. The initial goal of aiding the service experts in identifying issues rapidly and in a timely manner before users do has been achieved, and successful early identification of anomalous events has been demonstrated in many cases and on a daily basis since the system has been set up.

## Data Availability

The raw data supporting the conclusion of this article will be made available by the authors, without undue reservation. Please contact them directly asking for access.

## Footnotes

1
https://www.elastic.co/what-is/elasticsearch.

2
https://www.elastic.co.

3 see https://www.tensorflow.org/.

4 see: https://www.cs.toronto.edu/∼tijmen/csc321/slides/lecture_slides_lec6.pdf.
